# Human Click-Based Echolocation of Distance: Superfine Acuity and Dynamic Clicking Behaviour

**DOI:** 10.1007/s10162-019-00728-0

**Published:** 2019-07-08

**Authors:** Lore Thaler, H. P. J. C. De Vos, D. Kish, M. Antoniou, C. J. Baker, M. C. J. Hornikx

**Affiliations:** 1 Department of Psychology, Durham University, Science Site, South Road, Durham, DH1 3LE UK; 2 Eindhoven University of Technology, Eindhoven, The Netherlands; 3 World Access for the Blind, Placentia, CA USA; 4 Department of Electronic Electrical and Systems Engineering, University of Birmingham, Birmingham, UK

**Keywords:** sonar, audition, blindness, SNR, signal design

## Abstract

Some people who are blind have trained themselves in echolocation using mouth clicks. Here, we provide the first report of psychophysical and clicking data during echolocation of distance from a group of 8 blind people with experience in mouth click-based echolocation (daily use for > 3 years). We found that experienced echolocators can detect changes in distance of 3 cm at a reference distance of 50 cm, and a change of 7 cm at a reference distance of 150 cm, regardless of object size (i.e. 28.5 cm vs. 80 cm diameter disk). Participants made mouth clicks that were more intense and they made more clicks for weaker reflectors (i.e. same object at farther distance, or smaller object at same distance), but number and intensity of clicks were adjusted independently from one another. The acuity we found is better than previous estimates based on samples of sighted participants without experience in echolocation or individual experienced participants (i.e. single blind echolocators tested) and highlights adaptation of the perceptual system in blind human echolocators. Further, the dynamic adaptive clicking behaviour we observed suggests that number and intensity of emissions serve separate functions to increase SNR. The data may serve as an inspiration for low-cost (i.e. non-array based) artificial ‘cognitive’ sonar and radar systems, i.e. signal design, adaptive pulse repetition rate and intensity. It will also be useful for instruction and guidance for new users of echolocation.

## **INTRODUCTION**

Echolocation is the ability to use reflected sound to get spatial information from the environment. It is possibly best known from bats, but some people who are blind have also trained themselves in active echolocation using mouth clicks (Kolarik et al. 2014; Stoffregen and Pittenger 1995; Thaler and Goodale 2016). Compared to typical artificial sonar and radar systems that may use hundreds of emitters and receivers, human echolocators have only one emitter (mouth) and two receivers (ears). Yet, previous work has shown that this ‘simple’ apparatus enables them to perform accurately in tasks that range from localization in azimuth, identification of materials, shape or size, to tasks that require orienting in space (Kolarik et al. 2014; Stoffregen and Pittenger 1995; Thaler and Goodale 2016). Whilst there have been previous investigations into the acuity of human echolocation with respect to distance (Kellogg 1962; Schoernich et al. 2012; Tonelli et al. 2016; Wallmeier and Wiegrebe 2014a), these studies were limited to individual participants (i.e. tested only single blind echolocators) or samples of sighted participants not experienced in echolocation. Data on the actual clicking behaviour during echolocation of distance is missing also. Importantly, learning about performance possibilities (but also limits) and about dynamics of sampling behaviour are essential for understanding the principles underlying echolocation performance.

With respect to acuity in depth, previous reports into human echolocation of distance were based on samples of sighted participants not experienced in echolocation and/or individual experienced participants (Kellogg 1962; Schoernich et al. 2012; Tonelli et al. 2016; Wallmeier and Wiegrebe 2014a). Those reports found that distance discrimination in human echolocation scales with reflector distance, such that people are better at detecting changes in the distance of reflector at closer ranges. In single blind individuals, best discrimination thresholds reported were ~ 10 cm at a reference distance of 60 cm (i.e. 16.7 % change) (Kellogg 1962; method of constant stimuli; the emission signal used was not reported) or 9.3 cm at a reference distance of 75 cm or 19 cm at a reference distance of 200 cm (i.e. 12.4 % and 9.5 % change, respectively) (Wallmeier and Wiegrebe 2014a; adaptive staircase procedure; click emissions). In samples of sighted participants without experience in echolocation but who had been trained in click-based distance echolocation for a specific experiment, best average thresholds are around 20 % across various distances (Schoernich et al. 2012; Wallmeier and Wiegrebe 2014a; adaptive staircase procedure; click emissions). Tonelli et al. (2016) also trained and tested a sample of sighted participants. They presented participants with an object at one distance at a time (30–150 cm in 30 cm steps) and participants indicated which distance had been presented by choosing an integer number 1–5. Their behavioural measure of precision was distance error, with best average precision around 17 % across various distances. It is at present unclear to what level these performance measures are representative for performance in experienced human echolocators using mouth clicks.

With respect to clicking behaviour during echolocation, previous research has shown that human echolocators adapt their clicking behaviour (i.e. increase number of clicks and intensity of the clicks) for weaker reflectors (Thaler et al. 2018). Whilst this previous work investigated reflection strength and clicking behaviour as a function of reflector location (e.g. front vs. side vs. back of the echolocator), it is the case that farther away reflectors will also return weaker echoes. As such, we might expect to find dynamic adaptive behaviour in this situation as well. Indeed, research in bats shows that bats increase the intensity of their calls when they are farther away from a reflector, and it has been suggested that increases in emission intensity serve to increase echo strength (Hiryu et al. 2007).

It is important to note for echolocation of finitely sized reflectors that as the distance of a reflector increases or decreases, the acoustic projection of the reflector also becomes smaller or larger. As such, for the echolocation of the distance of finitely sized reflectors, it is important to measure resolution of distance independent of reflector size. Thus, in the current study, we not only used the same sized reflector at different distances, but we also introduced a differently sized reflector at the same distance. In this way, we could test if the same resolution in depth is achieved regardless of reflector size.

## **METHODS**

The experiment was conducted following the British Psychological Society (BPS) code of practice and according to the World Medical Organization Declaration of Helsinki. All procedures had been approved by the Durham University Department of Psychology ethics committee (REF 14/13). Participants volunteered to take part in the study. Information and consent forms were provided in an accessible format, and we obtained informed consent from all participants. The current study had been conducted in the same series of work and with the same participants and facilities as one of our previous reports (Thaler et al. 2018). As such, where appropriate, we refer to method details to our previous report. Stimulus presentation and behavioural and acoustic analyses were done using Matlab (The Mathworks, Natick, USA) and custom written routines. Statistical analyses were carried out in SPSSv22.

### Participants

Eight blind participants with experience in echolocation took part in the experiment. The same participants had also taken part in one of our previous studies (Thaler et al. 2018) and all details have been described in that report. For completeness, we reproduce participant details in Table 1.

**Table 1:** Details of participants who took part in the study

Participant ID	Gender	Age at time of testing	Cause of vision impairment	Severity of vision impairment at time of testing	Age at onset of vision impairment	Age at start of using mouth click-based echolocation
S1	Male	53	Optic nerve compression	Right eye total blindness; left eye bright light detection (tested with blindfold)	5 years	43 years
S2	Female	41	Leber’s congenital amaurosis	Total blindness	Birth	31 years
S3	Male	49	Retinoblastoma	Total blindness	Birth; enucleation at 1 year	< 3 years
S4	Male	33	Optic nerve atrophy	Total	14 years	15 years
S5	Male	56	Retinal detachment	Bright light detection (tested with blindfold)	Birth	6 years
S6	Male	43	Leber’s congenital amaurosis	Bright light detection right eye; total blindness left eye; (tested with blindfold)	Birth	33 years
S7	Male	34	Glaucoma	Total blindness	Gradual loss since birth	12 years
S8	Male	32	Optic nerve atrophy	Bright light detection (tested with blindfold)	8 years	29 years

### Set-up and Apparatus

**Fig. 1:**
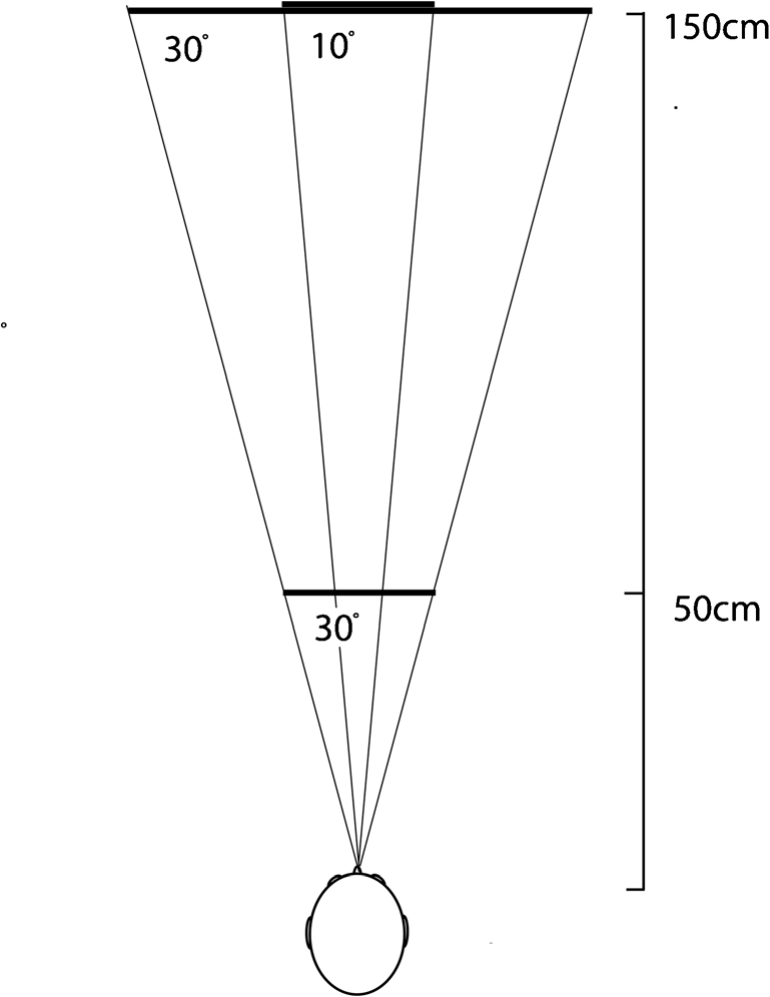
Sketch of the experimental set-up as seen from above. Reflectors were circular disks made from 5 mm thick wood (28.5 cm or 80 cm diameter) presented one at a time in front of the participant around a reference distance of either 50 cm (28.5 cm diameter only) or 150 cm (both 28.5 cm and 80 cm diameter). Drawn for illustration are the acoustic angles comprised by the reflectors at the various reference distances. The acoustic projection of the 80 cm disk at 150 and of the 28.5 cm disk at 50 cm was 30°. The acoustic projection of the 28.5 disk at 150 cm was 10°. Each reference distance was tested separately, but we have drawn reflectors at each distance for illustration. Angles and distances are drawn proportional

The work was conducted in a 2.9 m × 4.2 m × 4.9 m noise-insulated and echo-dampened room (walls and ceiling lined with foam wedges with cut-off frequency 315 Hz; floor covered with foam baffles, noise floor 24dBA). Participants stood in the centre of the room. Tactile markers were used to allow participants to reliably place their head at the same position throughout a trial, whilst not impeding movements of the mouth for clicking. Reflectors to be echolocated were wooden disks (28.5 cm or 80 cm diameter) presented one at a time in front of the participant around a reference distance of either 50 cm (28.5 cm diameter only) or 150 cm (both 28.5 cm and 80 cm diameter). Thus, at 50 cm, the 28.5-cm disk subtended 30° of acoustic angle, and at 150 cm, the 80-cm disk also comprised 30° of acoustic angle, whilst the 28.5 cm diameter at 150 cm disk comprised 10° of acoustic angle. On each trial, the reflector was first presented at the reference distance, and subsequently at a comparison distance that was either closer or farther away than the reference distance (following an adaptive staircase procedure). Reflectors were presented facing the echolocators with the centre placed at mouth level. Figure 1 illustrates the set-up. We made recordings of all testing sessions with a digital recorder (TASCAM DR100-MKII; TEAC Corporation, Japan; 24bit and 96 kHz) and with microphones (DPA SMK-SC4060 miniature microphones; 4 mm diameter; DPA microphones, Denmark) placed on either side of the participant’s head slightly in front and on top of the tragus.

### General Task and Procedure

Participants placed their head in the centre of the room facing straight ahead. The head had to be kept straight ahead on the tactile marker for the whole duration of a trial. To determine distance discrimination thresholds, we employed a 2-interval-2-alternative-forced-choice adaptive staircase method. The participant’s task on every trial was to determine whether a reflector at a test distance was located closer or of a reflector at a reference distance (either 50 cm or 150 cm). Presentation was sequentially, such that the reflector was always presented first at the reference distance and then at the test distance.

To minimize the possibility of procedural bias, two intertwined staircases were used that approached each reference distance from closer or farther away (for 50 cm reference, these were − 20 or 20 cm starting value, respectively, and for the 150 cm distance, they were 60 and − 60 cm). Presentation order of staircases was pseudo-random such that one staircase would not run for more than four consecutive trials. The distance difference between test and reference on each trial was determined adaptively. In the first two trials, we used the stochastic approximation by Robbins-Monro (Robbins and Monro 1951):

$$ {x}_{n+1}={x}_n-\frac{c}{n\left({z}_n-\phi \right)} $$

where *n* is the number of the current trial, *x* the value of the stimulus (i.e. the distance of disk during test with respect to reference) and *c* the initial step size (set at 20 and 60 for the 50 and 150 reference distances, respectively), *ϕ* is the probability of responding in a correct or incorrect way with respect to the corresponding staircase (0.5 in our paradigm) and *z* defines if the response was correct (1) or incorrect (0), referring to the corresponding staircase (e.g. ‘closer’ is correct for the closer- and incorrect for the farther-starting staircase). For subsequent trials, we used the accelerated stochastic approximation by Kesten (1958):

$$ {x}_{n+1}={x}_n-\frac{c}{\left(2+m\right)\left({z}_n-\phi \right)} $$

which includes *m* for the number of changes in the response category, i.e. *m* increased by one when the response switched from closer to farther, or vice versa, in one staircase. The test was terminated either when the participant’s responses had reversed from closer to farther or vice versa eight times within each staircase. One session took at most 45 min to complete. There was a minimum of 15 min break in between sessions.

At the beginning of each trial, the participant would block their ears and hum. Then, the experimenter would place the reflector at the reference position. Then, the experimenter retracted to the back of the room, behind the participant. Once the experimenter was at the back of the room, they slightly tapped the foot of the participant (with a long cane). On that signal, the participant unblocked their ears and echolocated until they had a good sense of where the reflector was located (~ 6 s). Then, the participant would block their ears and hum again. The experimenter would then place the reflector at the test distance. After the reflector had been placed, the experimenter would again retract to the back of the room and signal the participant with a foot tap. The participant would then echolocate until they had a good sense of where the reflector was located (~ 6 s). Then, the participant would state whether the test was located closer or farther with respect to the reference. The experimenter would enter the response into a computer keyboard. The computer (placed in a different room) would then calculate the test position for the next trial, and display it on a monitor in the back of the testing room. During experimental trials, no feedback was given. The reason we refrained from giving feedback was that we did not want to bias participants’ behaviour in any way, and we also did not want to cause frustration. Whilst in the beginning of each staircase, i.e. for larger distance differences, perceptual differences and thus decision criteria were very clear for all participants, as the staircases progressed and distance differences became smaller (and in particular near threshold), the ‘correct’ response was expected to be less obvious and at times, all participants could do is guess.

Before the experiment started, the experimenter explained the task and procedure to the participant, and the participant completed three practice trials. During practice trials, which used the starting values for each staircase and as such were easy to perceive by all participants, the experimenter gave feedback. In addition, the participant was told that it might become increasingly more difficult to determine the position of the test with respect to the reference, and that this was a consequence of the procedure used. The participant was told that if they were uncertain about the position of the test with respect to the reference, they should respond with their ‘best guess’. The participant was also asked to keep their head stationary straight ahead placed on the tactile marker during presentation of the reflectors and in between presentation of the reference and the test. We instructed participants to give a response whenever they felt they were ready to do so (i.e. there was no limit on trial duration).

### Data Analysis

#### Behaviour

Psychophysical performance was measured by fitting two parameter sigmoid curves of the form $F=\frac{1}{1+\exp \left(-\frac{x-a}{b}\right)}$ to the data for each participant and test and then using these to compute thresholds and bias. Parameters were estimated by performing a non-linear least squares fit with a trust-region algorithm implemented in the Matlab optimization toolbox. Curves were fitted separately for each participant and test. To compute thresholds, we first determined those points on the curve where the probability to judge a reflector as ‘closer’ was either .25 or .75, and we then computed the average of the absolute values. To compute bias, we determined the point on the curve where the probability to judge a reflector as ‘closer’ was .5.

#### Acoustics

To characterize participants’ clicking behaviour, we analysed recorded sound files for each participant. We analysed the numbers of clicks made for each trial, click duration, intensity, inter-click intervals (ICIs) and click power spectra, as well as power spectral centroid, and bandwidth based on power spectra. We also computed RDLD (i.e. level difference between reflected and direct sound as measured at the ear) (Pelegrín-García and Rychtáriková 2016), echo intensity and echo power spectra. This was done to characterize participant’s echo-acoustic sensitivity. Please note that the concept of RDLD is related to the concept of target strength, as used in dolphin or bat echolocation, and which Au (1993) defined as the ratio (in dB) of the echo intensity measured 1 m from the target to the intensity of the incident signal (i.e. the emission) measured at the target. The number of clicks for each trial was determined visually and aurally by visual and aural screening of the sound files. During this process, clicks were also isolated from intermittent speech and other background noise (e.g. coughing, swallowing, etc.) for further analysis. Click duration was computed as the time from click onset to offset. To obtain onset and offset, we first computed the click envelope as the absolute value of signal and smoothing it with a moving average using a 0.42-ms-duration window. Click onset was determined as the first point where envelope value exceeded 5 % (-26 dB) of the maximum. The offset was determined as the first point where the envelope dropped to 5 % (− 26 dB) of the maximum. Click duration could only be computed for a fraction of all clicks for the condition where the reference distance was at 50 cm, because for large numbers of trials, the click duration exceeded the echo onset, meaning that the click and echo overlapped. Thus, to avoid bias in the calculation of average duration, we calculated minimum duration for clicks at all reference distances, but average click duration only for 150 cm reference distances. Click intensity was computed as root mean square (RMS) intensity of clicks for 2.6 ms from the onset of the click. Clicks were truncated at this time to avoid biasing the analysis due to overlap between click and echo. To characterize spectral content of clicks, we computed each click’s power spectrum (based on the same 2.6 ms click duration for all conditions) and then determined the power spectral centroid, and bandwidth (using a 25-dB drop relative to peak (Arditi et al. 2015), and using the powerbw.m function implemented in the Matlab signal processing toolbox) for each trial, and then averaged across trials for each distance. To compute RDLD, we determined click and echo RMS intensity, but only for those sounds where echo and click were separated in time, and then took the difference. Since reflectors had been presented straight ahead, RDLD and echo intensity were averaged across right and left channels. The echo was detected by windowing of the sound at the expected time of the echo (since the reflector had been placed at various distances), and determining RMS intensity using the same method as used for clicks. To characterize spectral content of echoes, we computed their power spectrum using the same method as used for clicks. For one participant at the 50-cm reference position, echo acoustics could not be computed because this participant’s click durations always exceeded echo onset time. For the other participants and conditions, there were sufficient ‘clean’ echo samples so that RDLD could be computed. Table 2 provides numbers of sound files used for calculation of RDLD, echo intensity and spectrum for the various conditions.

**Table 2:** Numbers of sounds used in each condition for calculations of RDLD and echo intensity

Participant	28.5 at 50 cm	28.5 at 150 cm	80 at 150 cm
A	18	279	177
B	14	230	184
C	112	359	242
D	–	181	152
E	50	236	183
F	52	250	153
G	9	134	114
H	13	228	219

#### Statistical Analysis

To investigate effects of the different conditions on thresholds and clicking behaviour, we subjected data to repeated measures ANOVA. Post hoc pairwise comparisons were done using *t* tests (paired samples). For all analyses, statistical significance was determined using an alpha level of .05. Greenhouse Geisser correction was applied if the sphericity assumption could not be upheld.

## **RESULTS**

### Psychophysical Performance

**Fig. 2:**
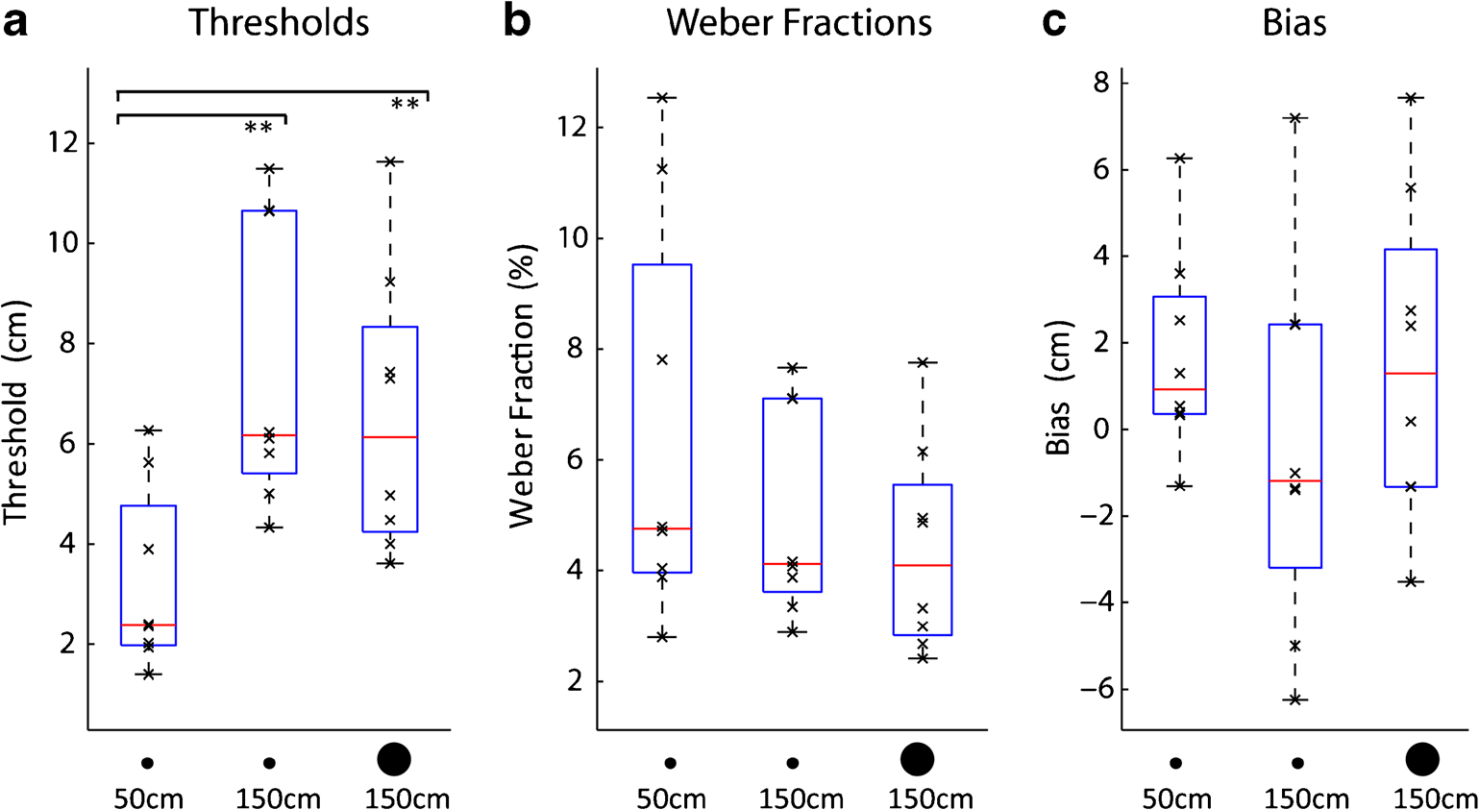
Measures of psychophysical performance. (a) Thresholds (b) Weber Fractions (c) Bias. Box and Whisker plots with red horizontal bars and lower/upper box boundaries representing median and 25th/75th percentile, respectively. Whiskers extend to 1.5 IQR, drawn back to the closest data point. Black crosses denote data from individual participants. Asterisks indicate results of paired *t* tests. **p < .01; For details, see main text

Figure 2a, b, c shows participant performance in terms of threshold, Weber fraction and bias, respectively, in the different conditions. People’s ability to echolocate distance was remarkably good. Expressed in thresholds (Fig. 2a), they were able to detect a ~ 3 cm change at the 50-cm reference distance and a 7-cm change at 150 cm reference distance, regardless of disk size. Consistent with this observation, the results of the statistical analysis showed a significant effect of distance on thresholds (*F*(2,14) = 8.418; *p* = .004; *η*^2^_*p*_ = .546), with post hoc tests showing that thresholds only differed between the 50 cm and 150 cm reference distances (28.5 at 50 cm vs. 28.5 at 150: *t*(7) = 4.372; *p* = .003; 28.5 at 50 cm vs. 80 at 150: *t*(7) = 3.592; *p* = .009), but not between the small and large reflector at 150 cm (*t*(7) = .712; *p* = .5). When thresholds were expressed as percentages, i.e. Weber fractions (Fig. 2b), participants were able to determine a 5 % change on average across all conditions, and there was no significant difference across conditions (*F*(2,14) = 1.886; *p* = .188; *η*^2^_p_ = .212). People’s bias (Fig. 2c) was not statistically different from zero in any of the conditions (28.5 at 50 cm: mean 1.71; SD 2.37; *t*(7) = 2.039; *p* = .081; 28.5 at 150 cm: mean −.372; SD 4.33; *t*(7) = −.243; *p* = .815; 80 at 150: mean 1.55; SD 3.78; *t*(7) = 1.163; *p* = .283), and did not differ across conditions (*F*(2,14) = 3.514; *p* = .058; *η*^2^_p_ = .34). Thus, participants’ accuracy (bias) was unaffected by changes in their precision (threshold) in our experiment.

Our estimate of echolocation acuity in depth measured through thresholds is better than previous best estimates in samples of sighted participants (JNDs ~ 20 %, Schoernich et al. 2012; Wallmeier and Wiegrebe 2014a; JNDs ~17 %, Tonelli et al. 2016) and also better than best thresholds for individual experienced participants reported to date (JNDs 16.7 %; Kellogg 1962) or 12.4 % and 9.5 % (Wallmeier and Wiegrebe 2014a).

### Clicking Behaviour

**Fig. 3:**
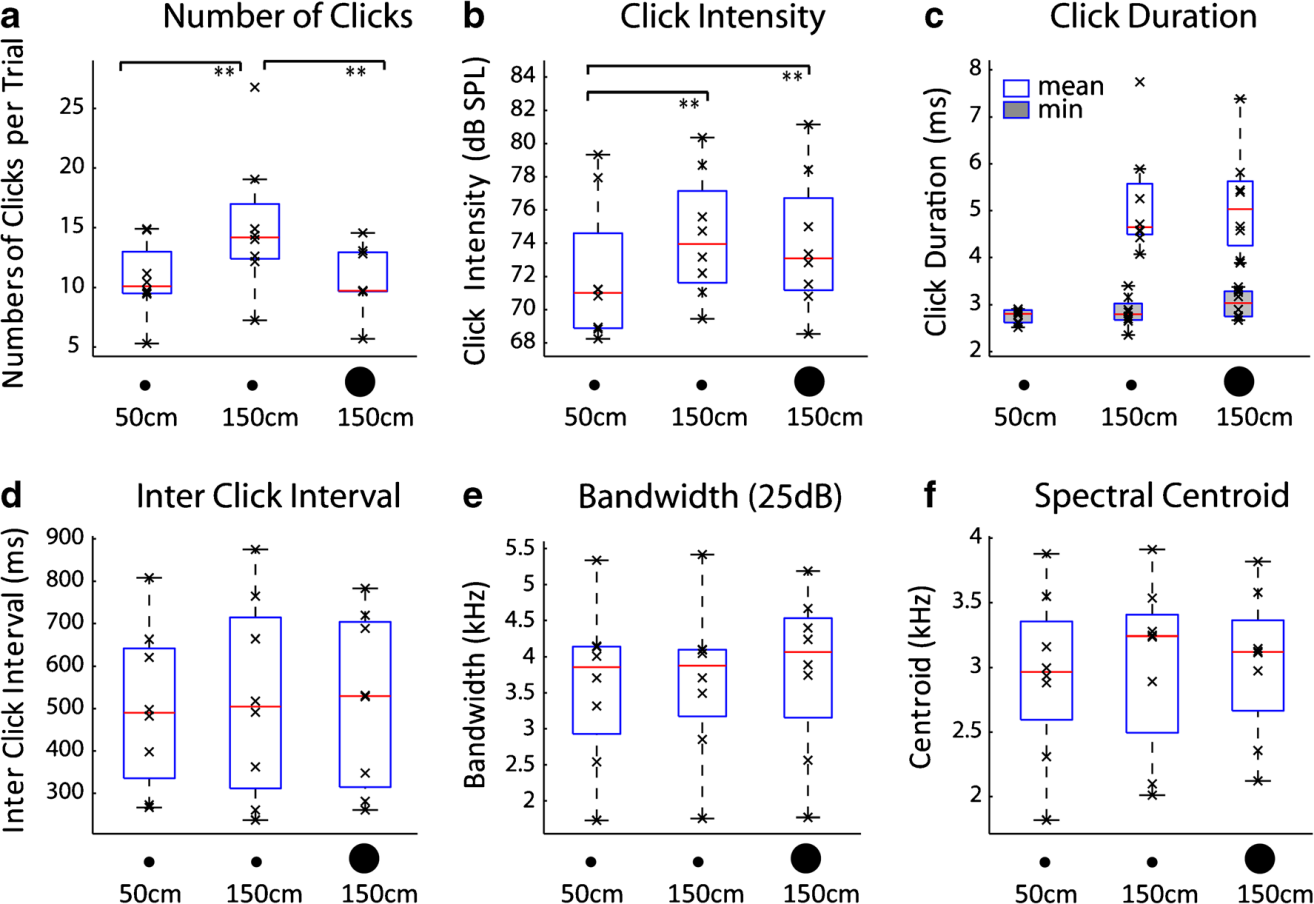
Measures of clicking behaviour. (a) Number of Clicks (b) Click Intensity (c) Click Duration (d) Inter Click Interval (e) Bandwidth (f) Spectral Centroid. Box and Whisker plots with red horizontal bars and lower/upper box boundaries representing median and 25th/75th percentile, respectively. Whiskers extend to 1.5 IQR, drawn back to the closest data point. Black crosses denote data from individual participants. Asterisks indicate results of paired *t* tests. **p < .01; For details, see main text

**Fig. 4:**
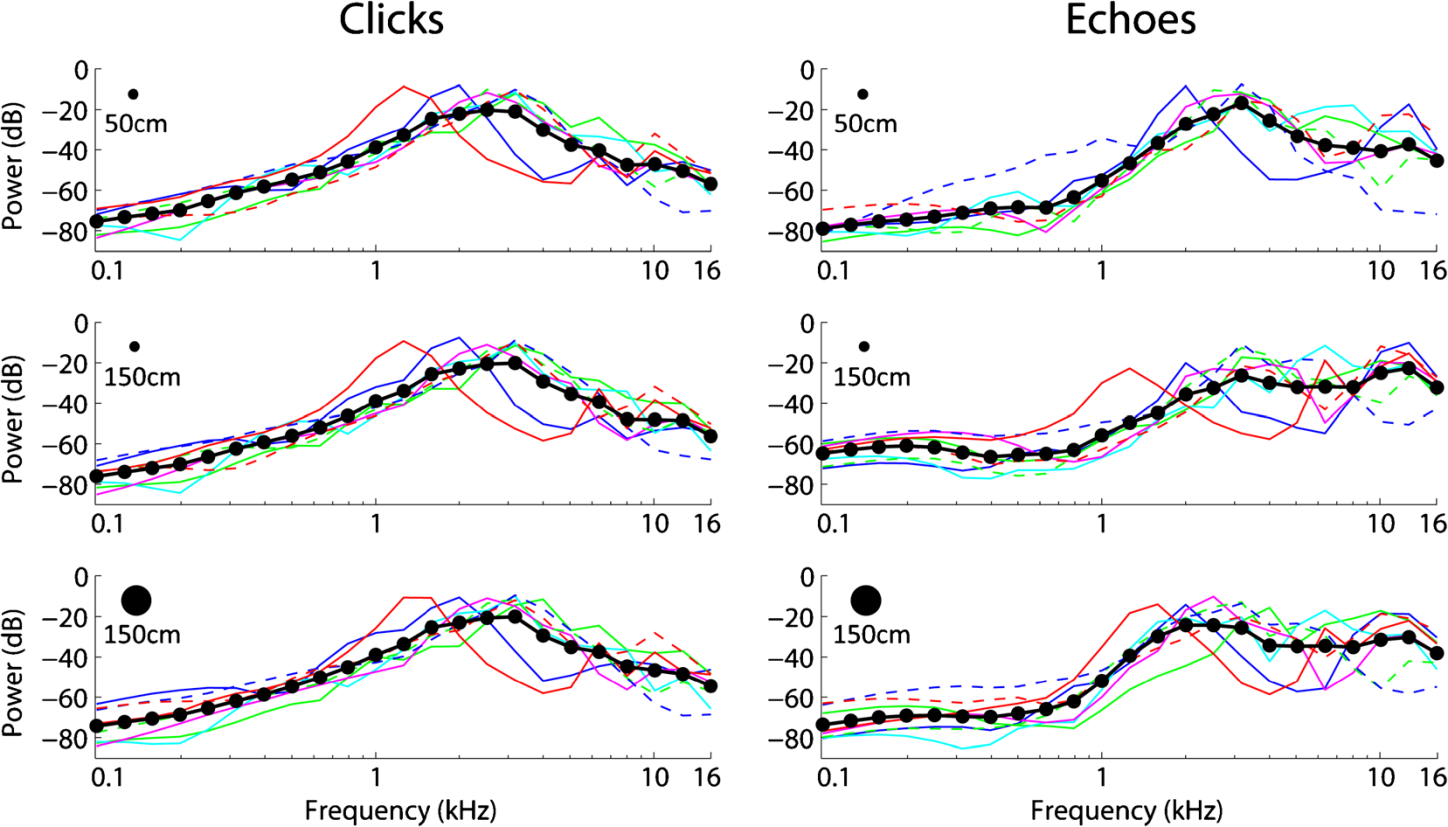
Power Spectra (1/3 Octave Bands with respect to total power) for clicks (left column) and echoes (right column) for the different conditions. Thin lines denote data for individual participants, where the same line colours and types denote data from the same participant across conditions. Thick lines and symbols denote the average across participants. For one participant (red lines), echo spectrum could not be computed at the 50-cm reference position because this participant’s click durations always exceeded echo onset time (see methods). Spectral content of clicks remains unchanged across conditions. Echoes show a shift towards higher spectral frequencies as compared to clicks, more so for smaller targets

Figure 3a–f shows measures of peoples’ clicking behaviour. Regarding the number of clicks people make (Fig. 3a), people changed the number of their clicks in response to a change in reflector distance and size (*F*(1.119,7.833) = 13.283; *p* = .006; *η*^2^_p_ = .655). Specifically, they increased the number of their clicks when the 28.5-cm reflector was moved from 50 to 150 cm reference distance (*t*(7) = 3.86; *p* = .006), and decreased the number of their clicks when the reference reflector at 150 cm increased in size from 28.5 to 80 cm (*t*(7) = 3.6; *p* = .009), whilst there was no difference in terms of number of clicks between the 28.5 cm reflector at 50 cm and the 80 cm at 150 cm (*t*(7) = .159; *p* = .878). Regarding the intensity of their clicks (Fig. 3b), people change the intensity of their clicks as a function of reflector distance (*F*(2,14) = 9.799; *p* = .002; *η*^2^_p_ = .583). Specifically, they increased the intensity as distance increased from 50 to 150 cm (28.5 at 50 cm vs. 28.5 at 150 cm: *t*(7) = 3.613; *p* = .009; 28.5 at 50 cm vs. 80 cm at 150 cm: *t*(7) = 4.387; *p* = .003), but intensity was the same for the 28.5 and 80 cm reflectors at 150 cm (*t*(7) = .777; *p* = .463). Click duration, inter-click interval click, bandwidth and click spectral centroid did not change across conditions (Fig. 3c–f). Consistent with lack of change in terms of bandwidth and power spectral centroid, power spectral density functions of clicks stay the same as well (Fig. 4 left column). The spectro-temporal pattern of clicks that we measured here was similar to those reported previously (de Vos and Hornikx 2017; Thaler et al. 2017).

**Fig. 5:**
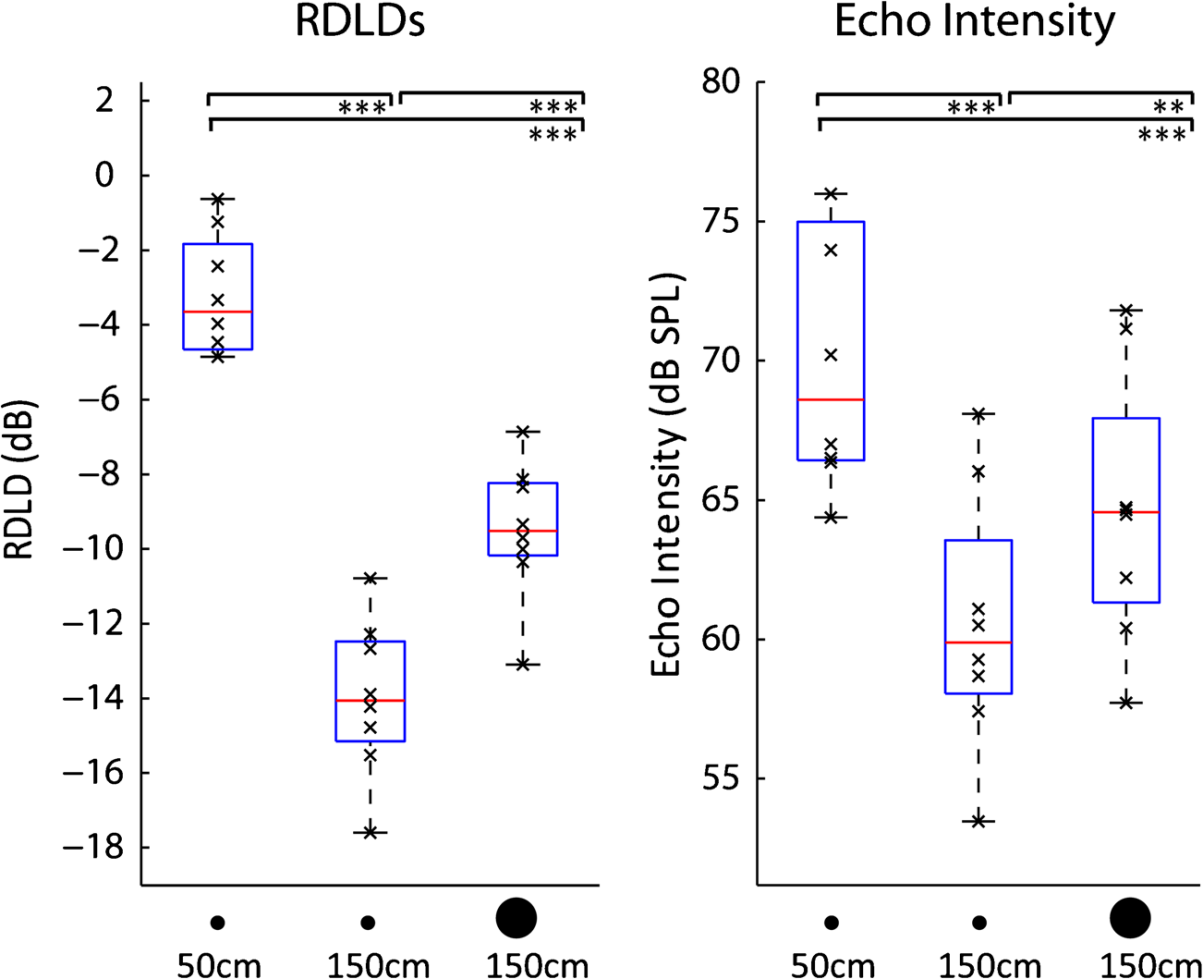
RDLDs (left panel) and echo intensity (right panel). Box and Whisker plots with red horizontal bars and lower/upper box boundaries representing median and 25th/75th percentile, respectively. Whiskers extend to 1.5 IQR, drawn back to the closest data point. Black crosses denote data from individual participants. Asterisks indicate results of paired *t* tests. **p < .01; ***p < .001. RDLDs and echo intensity decrease at farther distances (i.e. 50 cm vs. 150 cm reference distance) and as the reflector decreases in size (i.e. 28.5 vs. 80 cm at 150 cm reference distance). For details, see main text

To further characterize the acoustics, we calculated power spectral density of echoes (Fig. 4, right column), the difference between reflected and direct sound, RDLDs, for the various conditions and echo intensity (Fig. 5). Please note that the concept of RDLD (i.e. level difference between reflected and direct sound as measured at the ear) is related to the concept of target strength, as used in dolphin or bat echolocation, and which Au (1993) defined as the ratio (in dB) of the echo intensity measured 1 m from the target to the intensity of the incident signal (i.e. the emission) measured at the target. With respect to spectral content of echoes, they show a shift towards higher spectral frequencies as compared to clicks. In addition, the shift towards higher spectral frequencies is more pronounced for smaller targets. This is expected based on the physical relationship between the size of a finite target (as used here) and wavelength of sound. Specifically, larger wavelengths and thus lower spatial frequencies will not be reflected as efficiently by smaller targets, so that we would expect to see a relative increase of higher frequencies. With respect to RDLDs, these change across conditions (*F*(2,12) = 98.851; *p* < .001; *η*^2^_p_ = .943), and indeed RDLDs drop as the 28.5-cm reflectors move from 50 to 150 cm reference distance (*t*(6) = 14.624; *p* < .001), and also as the reflector at the same 150 cm reference distances decreases in size from 80 to 28.5 cm (*t*(7) = 8.234; *p* < .001). This result is expected because smaller and more distant reflectors will return weaker echoes. Consequently, the same pattern of results can be seen for echo intensities that change across conditions (*F*(2,12) = 71.528; *p* < .001; *η*^2^_p_ = .923), and indeed echo intensity drops as the 28.5-cm reflectors move from 50 to 150 cm reference distance (*t*(6) = 13.089; *p* < .001), and also as the reflector at the same 150 cm reference distances decreases in size from 80 to 28.5 cm (*t*(7) = 5.99; *p* < .001). Considered together with the changes in clicking behaviour (Fig. 4), it therefore seems to be the case that participants adapt their click intensity and number of clicks to partially compensate for the distance or size-dependent changes in RDLD (or echo-intensity).

## **DISCUSSION**

Our results show that people who have experience in click-based echolocation are able to echolocate distance of a reflector with high acuity. Their thresholds, i.e. their ability to resolve a change in the distance of a reflector scaled with the reference distance, i.e. ~ 3 cm at 50 cm reference distance and ~ 7 cm at 150 cm reference distance, were the same for both of the tested reflector sizes. Performance expressed as a percentage of the reference distance (i.e. Weber fractions) was 5 % on average and did not differ across conditions. This level of perceptual performance is much better than what had previously been reported based on research in sighted people. Specifically, in samples of sighted participants who had been trained in a specific click-based distance echolocation task, and using adaptive staircase procedures similar to the ones we used here, best average thresholds across various distances were around 20 % (Schoernich et al. 2012; Wallmeier and Wiegrebe 2014a). Tonelli et al. (2016) also trained and tested a sample of sighted participants. They did not use an adaptive staircase procedure, but presented participants with one distance at a time (30–150 cm in 30 cm steps) and participants indicated which distance had been presented by choosing an integer number 1–5. Their behavioural measure of precision was distance error. The best average precision across various distances was around 17 %. Though comparisons of our data to these results is indirect (i.e. we did not test sighted participants in our experiment), it is clear that psychophysical performance in our sample of blind people with experience in echolocation is superior, and as such indicative of adaptation of the perceptual system in blind human echolocators.

In addition to showing high accuracy and precision in terms of distance perception in blind echolocators, our data also show that adaptive emission strategies are used during this task. Specifically, analyses of click acoustics and echo-acoustic reflections suggest that people change number of their clicks or intensity of their click as the strength of echo-acoustic reflection changed, i.e. participants change their behaviour to partially compensate for the distance (or size) dependent changes in RDLD or echo-intensity. We can compare our findings to other echolocation mammals, e.g. bats or dolphins, that also increase the intensity of their emissions for farther away (and thus weaker) reflectors (e.g. Hiryu et al. 2007; Linnenschmidt et al. 2012), raising the possibility that human echolocation of distance may be governed by similar principles as in dolphins or bats. Yet, some bat species may also shift spectro-temporal aspects of their calls (i.e. intensity, duration, spectrum, pulse rate) pending on the environmental conditions (e.g. Ghose and Moss 2006; Moss et al. 2006; Siemers and Schnitzler 2004; Surlykke and Moss 2000; Ulanovsky et al. 2004; Vespe et al. 2010; Tressler and Smotherman 2009), or they may adjust the direction and/or width of their sound beam when they lock onto a target (Yovel et al. 2010; Ghose and Moss 2003, 2006; Surlykke et al. 2009). Our current study did not find any evidence for changes in click duration, spectrum or pulse rate, similar to our previous findings in blind echolocators (Thaler et al. 2018). This does not rule out that these aspects might change in other contexts, however. For example, it is possible that changes in click spectrum or duration might be observed when echolocating farther distances (e.g. 20 m or more). Furthermore, the paradigm used did not require self-movement of the echolocators, or approach of a target, and it is possible that for this reason, we did not observe changes in inter-click interval, click duration or spectrum, that are typically observed in bats during target approach. Humans can of course adjust click direction by moving their head. Since head movements were not permitted in our study, we did not measure dynamic adjustments in terms of head rotation. Nonetheless, it has been shown that under certain conditions, human echolocation can be facilitated by head movement (Milne et al. 2014; Wallmeier and Wiegrebe 2014b). Based on our current results, we suggest that future work should characterize these movements with respect to echo-acoustic sampling.

Notably, in our study, the reflector size that was used did not affect people’s ability to detect changes in the distance of the reflector, but it did affect people’s clicking behaviour, suggesting that people achieved the same level of performance for the two target sizes, but that they had to ‘work harder’ for the smaller target at the same distance. Specifically, people made more clicks for the 28.5 cm as compared to the 80 cm reflector, both at the same 150 cm reference distance. Notably, the intensity of the clicks that was used for these two target sizes at the same 150 cm reference distance was the same, and was about 74 dB SPL on average. Previous measurements of click intensity using the same set-up and microphone positioning but a different task (Thaler et al. 2018) had measured clicks as loud as 82 dB SPL on average, so that in principle it would have been possible for participants to make louder clicks (the same applies to click intensities based on peak values which were as loud as 93 dB SPL in our previous study, as compared to 84 dB SPL in our current report). The fact that they did not do so suggests that increasing intensity of clicks was not an adaptive behaviour to compensate for the change in target size we had made. In contrast, intensity of clicks was increased as targets were placed farther away, i.e. from 50 to 150 cm, and the increase in intensity was the same for the 28.5 and the 80 cm target size. In their entirety, therefore, the data suggest that echolocators independently adjust the number and the intensity of their clicks to compensate for weaker target reflectors, implying that these two strategies serve different functions in order to increase SNR.

Increasing the intensity of clicks leads to an increase in echo intensity. Therefore, just as in our previous report (Thaler et al. 2018), we conclude that it is likely that people (just like bats (Hiryu et al. 2007; Tressler and Smotherman 2009) increased click intensity to increase signal to noise ratio (SNR), where the signal is the echo and noise is residual ambient noise and/or noise intrinsic to the human auditory system. Close temporal proximity of clicks and echoes in our study (onset delay ~ 3 ms or ~ 9 ms) implies that detection of echoes will be affected by forward masking (of the echo by the emission) which sometimes goes into simultaneous masking (when click duration exceeds echo delay) (Moore 2012; Zwicker and Fastl 2013) and/or echo suppression (Litovsky et al. 1999; Wallach et al. 1949). The reason that an increase in click intensity is nonetheless a useful strategy to increase detection performance (by increasing SNR) is because of the non-linear behaviour of masking (Moore 2012; Zwicker and Fastl 2013). Increasing the number of clicks is expected to have the same purpose, i.e. to increase SNR. In fact, artificial systems and applications make use of this by averaging across multiple samples in order to increase signal to noise ratio. Just as in our previous report (Thaler et al. 2018), we therefore conclude that human echolocators must accumulate information from multiple samples over time. Importantly, the current report provides evidence that these two strategies (i.e. intensity or number of clicks) are adjusted independently.

Whilst our data describe performance and emission dynamics during echolocation of distance, they do not lend themselves to answering the question which acoustic cues may drive this behaviour. In principle, people could rely on the time delay between click and echo, the intensity of the echo, or spectral changes from other physical interference between click and echo or repetition pitch. The fact that people increase intensity of clicks for weaker reflections suggests that echo intensity is an important factor in performance. Yet, this does not imply that people rely on an intensity cue for determining distance. In fact, based on neural response properties, a more intense sound will also be lending itself to more reliable estimates of its onset or spectrum. Furthermore, if people were using only intensity as a cue, their threshold should only be about 20 % under ideal conditions (Wallmeier and Wiegrebe 2014a). Yet, performance in our study was better than this, suggesting that temporal and/or spectral factors play a role, too. With respect to temporal factors, participants in our study would have encountered a click-echo delay of ~ 3 ms for 50 cm and ~ 9 ms for 150 cm distances. In passive listening paradigms, thresholds for detecting a distinct echo vary widely, but are generally > 5 ms (Litovsky et al. 1999), possibly suggesting that participants may not have had access to explicit click-echo delays in our paradigm, at least for the 50-cm reference distance. Yet, the precedence effect is reduced in active echolocation, i.e. when people make their own clicks (Wallmeier et al. 2013). There is also the possibility that time integration windows as measured in sighted participants (which is the type of participant group the precedence effect literature is focused on) might not generalize to blind people and/or blind people with experience in echolocation. For example, it has been shown that blind people have a better ability than sighted people to resolve two 2500 Hz sounds occurring in rapid succession (Muchnik et al. 1991). In other words, a blind person might be able to hear two sounds rather than one when the two sounds are separated by a silent gap as short as 5 ms, whilst a sighted person may hear only a single merged sound (Table 2 in Muchnik et al. 1991). More systematic work is needed to determine which acoustic cues people may use for echolocation of distance. In this context, the data we present here can be used as a benchmark against which theoretical models can be compared.

Ranging accuracy in echolocating bats is better than what we observe here in people, i.e. bats have demonstrated millimetre (or even sub-millimetre) resolution (Moss and Schnitzler 1989). Yet, this can be understood considering that the emissions bats use contain much higher spectral frequencies (i.e. ultra sound), and may also be of much shorter duration, in particular for bats that use clicks (Surlykke and Moss 2000; Yovel et al. 2011).

Recordings in our study were made next to the tragus of each ear. Nonetheless, even though our measurements do not allow us to describe intensity of the click signal as measured at the mouth, our measurements are suited to quantify changes in transmitted click intensity across conditions. Specifically, even though changes in sound intensity measured at the ear can be due to changes either in intensity of the sound made at the mouth or changes in directionality of the sound, directionality of sounds can only be altered by changing the shape of the mouth, i.e. increasing mouth aperture. Importantly, however, changes in mouth aperture would also cause changes in spectral content of the clicks (Halkosaari et al. 2005; Monson et al. 2012. In our study, we did not observe any change in spectral content across conditions. As a consequence, changes in click intensity that we measured at the ear are likely due to changes in intensity of the clicks, rather than changes in directionality.

In conclusion, our data highlight ensonification strategies of blind human echolocators as well as adaptations that take place in their perceptual abilities. Using this model system to learn about adaptive strategies such as flexible pulse repetition rates or intensity will be helpful for developing low-cost (i.e. non-array based) artificial radar and sonar systems, because understanding in which situations humans employ one or the other method (i.e. increasing pulse rate vs. intensity) can serve as an inspiration for developing these systems. At present, only research-based radar systems are beginning to emerge that have the ability to independently adjust parameters such as pulse repetition rates, pulse lengths and intensity (Smith et al. 2016). Working with humans in this context has the advantage that talking to them facilitates instruction and measurements. Furthermore, echolocation is a useful skill for people who are blind. Learning about the possibilities (but also the limits) of human performance as well as learning about their adaptive strategies will be useful for instruction and guidance for new users.
